# The Effects of Coupling Factors on the Variable Loading Resistance of Plain-Woven Ultra-High Molecular Weight Polyethylene Fabric Composites

**DOI:** 10.3390/polym18070839

**Published:** 2026-03-30

**Authors:** Ziyan Zhou, Feilong Han, Bin Dong, Wen Zhai

**Affiliations:** 1 Shandong Institute of Non-Metallic Materials, Jinan 250031, China; 15927101046@163.com (Z.Z.); dongbin53@163.com (B.D.); 2 Tayho Advanced Materials Group Co., Ltd., Yantai 250031, China; hfl2440@163.com

**Keywords:** ballistic impact resistance, variable loads, PE plain-woven fabric/waterborne polyurethane composites, simulation analysis, relative weight analysis

## Abstract

Resin and interlayer properties play significant roles in the resistance to impact of fibre-reinforced polymer composites (FRPCs). To investigate the contribution of each factor within the coupled variables to the impact resistance ability of FRPCs, in this work, waterborne polyurethane (WPU) with different tensile elastic modulus, tear strength and bonding strength was obtained. To systematically evaluate the impact resistance and failure mechanisms of the composite materials under varying external loads, impact resistance tests, numerical simulations, and relative weight analysis were conducted. The relative weight analysis results quantified the individual contributions of these three factors to the overall energy absorption capacity across diverse loading conditions. The results indicated that with the increasing rate of the external loading, the resin modulus consistently contributed more significantly to energy absorption than tear strength of resin and interlayer strength, reaching up to 44.3%. In ballistic penetration tests, with the increase in resin modulus, the ballistic performance of PE/WPU laminates demonstrated an S-shaped downward trend. Composites prepared with more rigid matrix could lead to unsatisfactory interlayer damage. A more robust structure could result in fibre pull-out and breakage to a greater extent at the point of forced impact while less in the secondary affected area, presenting comparatively lower impact resistant performance.

## 1. Introduction

Life safety plays a crucial role in human development, as human bodies are constantly exposed to various forms of impact damage in extreme circumstances, such as car crashes, explosions and high-speed shrapnel [1]. The combination of fibre and resin has been recognized as an effective technique to address this issue [2,3,4,5,6]. Numerous studies have explored the advantages of utilizing protective composites made from the combination of fibre and resin, including the ability to withstand heavier impacts while being lighter in weight, having superior capacities to absorb energy [7,8,9,10]. Compared to metal armour, fibre-reinforced polymer composites (FRPCs) are more efficient at dissipating impact energy and protecting soldiers. Therefore, FRPCs are the preferred material for designing and manufacturing impact-resistant protection equipment [11].

FRPCs are typically composed of fibre reinforcement and resin matrix. Fibres always serve as the primary load-bearing components under applied stress, while resin matrix not only influences the distribution and stress state of the fibres during loading, but also facilitates the rapid transfer of localized stress, thereby effectively dissipating impact energy. UHMWPE fibre, characterized by its high specific strength, high specific modulus, low density and hydrophobicity, has been widely used in FRPCs for ballistic protection [10,12]. WPU is known for its highly flexible elastic nature, characterized by a phase-separated structure and dynamic covalent bonds. Its excellent bending resistance and high peel strength confer exceptional impact and damage resistance to composites [13,14,15]. However, with the advancement and evolution of military weaponry, current protective materials are increasingly unable to meet the operational protection requirements for soldiers on the battlefield, and the next-generation alternative has not yet been established. Consequently, a more comprehensive investigation into existing materials is urgently warranted.

In practical applications, the impact resistance of FRPCs is influenced by multiple factors, many of which interact with each other. Han et al. [16] undertook a study on aramid-reinforced composites with diverse polycarbonate contents to explore the influence of resin contents on ballistic performance. The results demonstrated that the V50 value rose as the matrix content declined. Specifically, when the matrix content was reduced to 10%, the target plate presented the maximum V50 value of 550 m/s, along with a specific energy absorption (SEA) of 28.56 J∙m^2^/kg. Zhang et al. [17] fabricated UHMWPE laminates with diverse preformed hole structures and explored the influence of the number and spacing of these holes on the elastic properties of FRPCs. The results manifested that a specific quantity of preformed holes could effectively redistribute tensile stress at the edges of holes, thereby enhancing elastic resistance. Firouzi et al. [18] prepared UHMWPE targets by adjusting the laminating force. The results revealed that low layer pressures would reduce the ballistic penetration performance of the lamination. UHMWPE targets fabricated with resin under moulding pressure of 4 MPa exhibited the optimal penetration resistance, whereas further increase in moulding pressure did not lead to more significant improvement in the penetration-resistant performance. The individual contributions of coupled material parameters to the energy absorption of FRPCs under ballistic impact have yet to be clearly quantified. While substantial body of research had focused on varying single parameters—such as structures of preformed hole, content of resin, or moulding pressure—to assess their effects on ballistic performance, most studies did not account for the fact that modifying one parameter inevitably alters others. As a result, the synergistic interactions among these parameters and their combined influence on energy absorption remained poorly understood. This research addressed that gap by systematically exploring the coupled effects of three key material properties—tensile elastic modulus, tear strength and bonding strength—on the ballistic behaviour of composites. By quantifying how these interacting parameters collectively govern energy dissipation and damage mechanisms, this study offered new insights into the design of advanced composite systems for enhanced ballistic protection.

In this study, FRPCs were fabricated with resin of different modulus denoted as PE/WPU X, where X represented resin number (the tensile modulus of elasticity was classified from A to E in increasing order). To investigate the contribution ratios of modulus of resin, tear strength of resin, and interfacial bonding strength to the energy absorption capacity of FRPCs, as well as the influence of matrix properties on the energy absorption of FRPCs under varying external loading conditions, quasi-static tests, low-speed load environment assessments, high-speed load environment evaluations and numerical simulation experiments were conducted. The energy absorption values of the samples under varying external loads were systematically calculated and recorded. Relative weight method was employed to analyze the proportional influence of changes in resin modulus, resin tear strength, and interfacial strength on the energy absorption performance. The results of the relative weight analysis indicated that, across all tested external loading conditions, the tensile elastic modulus of the resin exerted the greatest influence on the energy absorption capacity of FRPCs, with its contribution becoming higher under high-speed impact conditions. Through the integration of testing and simulation, it was found that augmenting the interlayer binding force of the composites within a specific range could influence the ballistic performance of FRPCs greatly, leading to a result where the ballistic performance first increased and then decreased. Higher interlayer bonding force and matrix with higher modulus may lead to stress concentration and incomplete delamination of the composite material. Lower interlayer bonding force and more malleable matrix could exhibit a superior capacity to transfer stress and relatively clear stratification pattern, leading to increased secondary deformation, drawing, and breakage of the yarn, thereby enhancing the ballistics performance of the material. This study provided a theoretical basis for the structural design of composite materials used in body armour by revealing how the coupling effects of tensile strength, shear strength, and bonding strength influence ballistic performance. The findings enabled the synergistic optimization of these material parameters, offering the potential to reduce equipment weight while enhancing protective efficiency.

## 2. Materials and Methods

**Figure 1:**
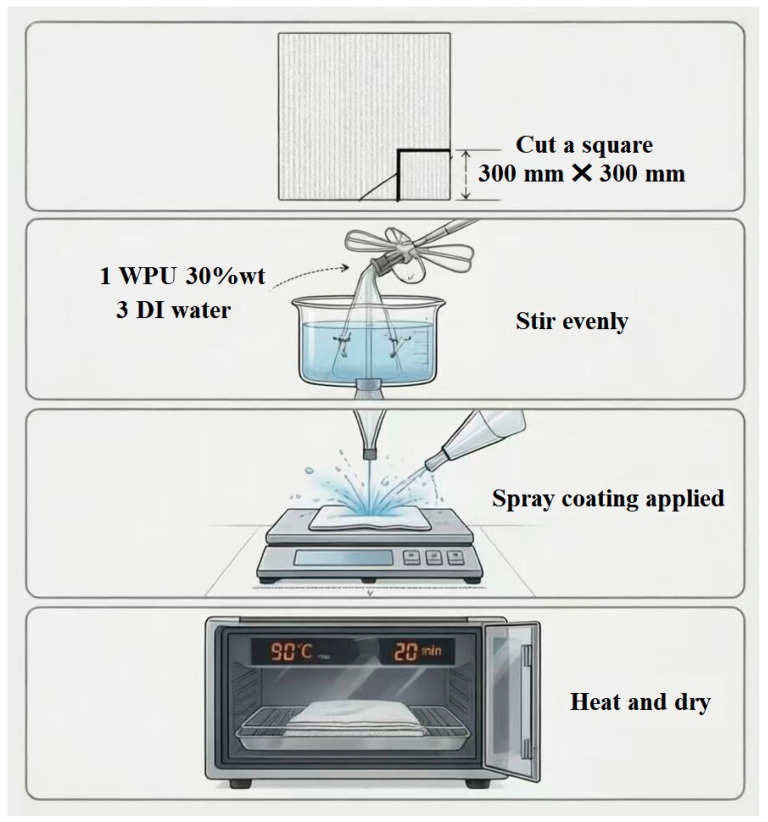
The preparation process of single-layer PE/WPU composite.

In this study, UHMWPE plain-woven fabric supplied by Zhongtai special equipment Inc., Changde, China, was used as reinforcement. The fabric had an areal density of 130 g/m^2^, denier of 800 and thread-count of 40 × 30 yarns per inch. Ultimate tensile strength of individual fibre was 3.4 GPa. WPU was supplied by Feidun New Materials Technology Co., Shanghai, China. Additionally, 300 mm × 300 mm UHMWPE plain-woven fabric sheets were prepared. The prepared resin was mixed with deionized water at a ratio of 1:3, and the diluted mixture was evenly applied onto the fabric pieces. During impregnation, the resin content was controlled within 20 ± 3%. The impregnated materials were then placed in an oven and heated at 90 °C for 20 min. The preparation process is shown in Figure 1.

Subsequently, the PE/WPU X laminates were fabricated in a hot-pressing machine, incorporating layers of UHMWPE composites. The UHMWPE layers were meticulously stacked on the hot-press machine, with a layer of tetrafluoroethylene film placed on both the upper and lower surfaces to prevent bonding. The metal mould was then heated from room temperature to 90 °C at a heating rate of 5 °C/min and kept at a pressure of 4 MPa for 2 min. The moulding pressure was regulated at 0–5 MPa three times within the first 5 min to evacuate any bubbles inside the laminates. Finally, the machine was turned off while maintaining pressure to prevent deformation, allowing the PE/WPU X laminates to cool naturally to room temperature.

**Figure 2:**
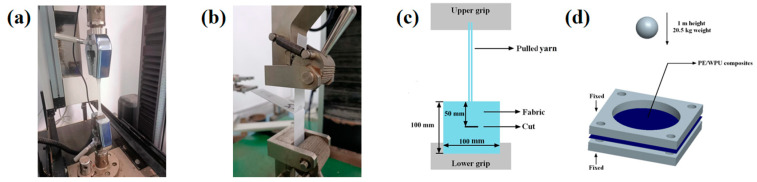
Experimental equipment of (**a**) quasi-static tensile test and (**b**) T-peel test. Schematic diagram of (**c**) yarn pull-out test and (**d**) low-velocity impact test.

Dynamic mechanical analysis of the resins was operated by TA Q800 instrument (TA Instruments, New Castle, DE, USA). The resins were fixed on a film tension clamp and scanned from −120 °C to 150 °C at a heating rate of 3 °C/min and a frequency of 10 Hz. According to ASTM D638-02a [19], the tensile properties of the WPU X were tested using Instron 5566 electronic universal machine with a stretching rate of 10 mm/min. The quasi-static mechanical properties of PE/WPU laminates were assessed by cutting 300 mm × 300 mm samples from the laminates, with 5 specimens selected for each test. Tensile properties of these PE/WPU X laminates were measured using a universal testing machine (Instron 5982, Instron corporation, Norwood, MA, USA) in accordance with ASTM D3039 [20] as shown in Figure 2a. T-peel tests were conducted using the Instron 5982, with the strength measured at a loading rate of 100 mm/min in accordance with ASTM D1876-08 [21]. The dimensions of the specimens were 130 mm × 20 mm × 2 mm, and a 65 mm × 20 mm polyimide film was inserted into each specimen to serve as an initial crack. Shape and gripper of the fixture were shown in Figure 2b.

FRPCs with same resin ratio were prepared using UHMWPE plain-woven fabric of the same surface density (130 g/m^2^). The tests aimed to study the pull-out behaviour of UHMWPE fibres from the matrix, considering the modulus of each resin, which ranged from 6.4 MPa to 18.2 MPa. The experiments were conducted on a universal tensile testing machine (Instron 5982). Four yarns of each sample were fixed to the upper grip of tensile testing machine, while parts below the incision were secured to the lower grip as shown in Figure 2c. The upper jaw ascended at a velocity of 50 mm/min, during which the force required to extract yarn from the fabric was measured. Low-velocity impact test was carried out by a falling dart type impact resistance tester (Instron CEAST 9350, Instron corporation, Norwood, MA, USA) according to ASTM D3763 [22]. After the fabric had been impregnated with resin and fully dried, it was cut into a size of 100 mm × 100 mm and fixed on the pneumatic clamping fixture. The total mass of the impactor and its accessories remained consistently at 20.5 kg, and the diameter was 20 mm. During the testing process, the impactor struck the fabric from a height of 1 m at an impact speed of 4.4 m/s, and the total impact energy was calculated to be 201.04 J. Energy absorption data were collected and documented using a pulse data acquisition system. Figure 2d presents a schematic illustration of the low-velocity test, where the four corners of the samples were secured to a fixture, leaving the central circular region uncovered beneath the drop hammer to absorb the impact. Four tests were carried out on each material to ensure the reliability of the results.

**Figure 3:**
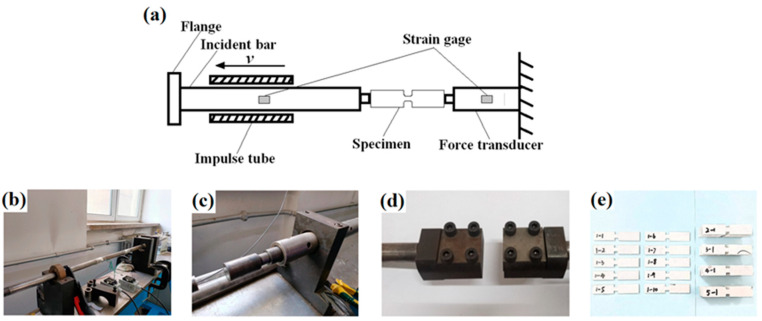
Equipment and sample drawings of HSR tests: (**a**) schematic diagram of HSR tensile test; (**b**–**d**) photos of HSR tensile apparatus; (**e**) HSR tensile test samples.

The HSR tensile test was conducted using PE/WPU X laminates, with the area density of each sample precisely controlled at 2.5 ± 0.1 kg/m^2^, and the samples were cut into the shape as depicted in Figure 3e. Figure 3a presents a schematic diagram of the HSR experiment, while Figure 3b provides an overview of the experimental setup. The incident rod and flange at the left end were designed to provide HSR kinetic energy (Figure 3c), and Figure 3d illustrates the configuration of the HSR tension fixture.

A concentric impact rod was wrapped outside the left rod and was subjected to a rated pressure by the hydraulic device to impact flange at the left end, while the right rod did not apply additional force. Strain gauges were installed on both sides of the rods to measure stress–strain curve of the experimental samples. Purpose of the experiment was to study the tensile properties of PE/WPU X under HSR conditions, thereby evaluating their ballistic performance for applications in soft bulletproof layers. It was assumed that the fibre specimen was in dynamic force equilibrium. According to one-dimensional wave propagation theory, one-dimensional strain and particle displacement in the incident rod could be given by the following formulas:

(1) $$\varepsilon =\frac{\partial u}{\partial x}$$

(2) $$u=f(x-c_{b}t)+g(x+c_{b}t)$$

(3) $$u\prime =c_{b}(-f\prime +g\prime )=c_{b}(-\varepsilon_{i}+\varepsilon_{r})$$

(4) $$\varepsilon \prime =-\frac{u\prime }{l_{s}}=\frac{c_{b}}{l_{s}}(\varepsilon_{i}-\varepsilon_{r})$$

where *u* was the axial displacement of the fibre, *f*(*x* − *c_b_t*) and *g*(*x* − *c_b_t*) were the waveform functions of incident and reflected waves, respectively. *c_b_* was a constant representing the wave speed in the rod, determined by the material of rod. $\varepsilon_{i}$ and $\varepsilon_{r}$ respectively denoted the incident strains and reflected strains. ls represented the sample length. Finally, the equation of strain with respect to t could be obtained as follows:

(5) $$\varepsilon =\int_{0}^{t}\frac{c_{b}}{l_{s}}(\varepsilon_{i}-\varepsilon_{r})d\tau$$

The PE/WPU ballistic limit velocity (V50) test was conducted in accordance with NATO STANAG 2920 [23]. The V50 ballistic test device comprised a 7.62 mm fragment simulation projectile launcher and a fragment simulation projectile (FSP) weighting 1.1 g and measuring 5.5 mm in diameter. During the ballistic test experiment, the distance between test frame and launcher was set at 5 m, with an incidence angle of 0° ± 5°. V50 was a critical indicator of ballistic performance, representing a target speed with a 50% probability of penetration. It was widely utilized for evaluating the ballistic performances of different materials. In this experiment, a 6-shot V50 method was employed, involving three shots resulting in full penetration while the other shots causing partial penetration within a scattering range of 38 m/s. The V50 value for each experimental target plate was determined by averaging its speed obtained from these tests. The results were analyzed using a non-destructive testing technique of X-Ray Computed Tomography (CT) scans to characterize delamination failure and study the failure mechanisms under ballistic impact. The CT scans were conducted on a CT ALPHA 600 operating at 450 kVp and 3300 μA X-ray. The X-AID Reconstruction system 2020 software was used to acquire 2320 × 2320 pixel projections (0.14 mm × 0.14 mm pixels) over 3040 projection angles. The projection images were reconstructed using a Feldkamp cone beam reconstruction algorithm into 0.152 mm voxels. The visualization and measurement processing were performed using Amire 2020 software. Scanning electron microscope (SEM) scanning was conducted on the fracture surface of the samples following both static tensile tests and HSR tensile tests. The GeminiSEM 300 instrument, Carl Zeiss AG, Baden-Württemberg, Germany, was utilized, operating at a scanning voltage of 5 kV and maintaining a distance of approximately 13 mm from the imaging surface of the sample to the objective lens.

In order to research the ballistic response of PE/WPU laminates with varying interlayer bonding strength, finite element models were established using ABAQUS/Explicit 2019 software. The numerical simulation employed the Lagrange method within ABAQUS/Explicit, facilitating an efficient reproduction of the dynamic loading process. The PE/WPU laminates were discretized using three-dimensional solid elements. A structured hexahedral mesh was employed, with eight-node linear brick elements (C3D8R) featuring reduced integration and hourglass control. Mesh refinement was applied locally in the vicinity of the projectile impact zone to enhance solution accuracy. The FSP was meshed with C3D8R elements, and rigid body constraints were introduced to the FSP, as no plastic deformation occurred during perforation. The FE models were developed as ¼-symmetry models to expedite calculation due to the structural symmetry of the FSP and laminates. The clay bottom Table 1, as well as the edges of the laminates and clay, were subjected to fixed boundary conditions. General contact with a friction coefficient of 0.23 was applied to the interface between the laminates. The material properties of the layers and interlaminar matrix were presented in Table 2 and Table 3. It is essential to validate whether perforation or non-perforation occurred in each impact test. For the same material, the velocity difference between the perforation and non-perforation cases should be maintained within 10 m/s, and the average of the two values was taken as the V50 ballistic penetration velocity of the material. The 3D Hasin failure criteria were employed to assess the initiation damage in the materials, defined as follows:

Fibre tension failure (*σ*_11_ > 0):

(6) $$d_{ft}={\left(\frac{\sigma_{11}}{X_{T}}\right)}^{2}+{\left(\frac{\tau_{12}}{S_{12}}\right)}^{2}+{\left(\frac{\tau_{13}}{S_{13}}\right)}^{2}$$

Fibre compression failure (*σ*_11_ < 0):

(7) $$d_{fc}={\left(\frac{\sigma_{11}}{X_{C}}\right)}^{2}$$

Matrix tension cracking (*σ*_22_ ≥ 0):

(8) $$d_{mt}={\left(\frac{\sigma_{22}}{Y_{T}}\right)}^{2}+{\left(\frac{\tau_{12}}{S_{12}}\right)}^{2}+{\left(\frac{\tau_{23}}{S_{23}}\right)}^{2}$$

Matrix compression cracking (*σ*_22_ < 0):

(9) $$d_{mc}={\left(\frac{\sigma_{22}}{2S_{12}}\right)}^{2}+(\frac{\sigma_{22}}{Y_{C}})\left[{\left(\frac{Y_{C}}{2S_{12}}\right)}^{2}-1\right]+{\left(\frac{\tau_{12}}{S_{12}}\right)}^{2}+{\left(\frac{\tau_{23}}{S_{23}}\right)}^{2}$$

In the equations above, *d_ft_*, *d_fc_*, *d_mt_*, and *d_mc_* represented the damage variates associated with each specific mode of damage. *σ*_11_, *σ*_22_, and *σ*_33_ denoted the stresses along the longitudinal, transverse, and vertical directions of the composites, respectively. Meanwhile, *σ*_12_, *σ*_13_, and *σ*_23_ represented the shear stresses. *X_T_*, *X_C_* and *Y_T_*, *Y_C_* signified the tensile strengths (*T*) and compressive strengths (*C*) in the warp (*X*) and weft (*Y*) directions of the composites. Additionally, *S*_12_, *S*_13_, and *S*_23_ indicated the longitudinal shear strength as well as two transverse shear strengths.

In this work, the cohesive elements utilized to characterize interlaminar matrix failure (i.e., delamination damage) were established based on a quadratic traction–separation law:

(10) $${\left(\frac{t_{1}}{t_{1}^{0}}\right)}^{2}+{\left(\frac{t_{2}}{t_{2}^{0}}\right)}^{2}+{\left(\frac{t_{3}}{t_{3}^{0}}\right)}^{2}=1$$

where *t_i_* (i = 1, 2, 3) was the interface stresses in the normal and shear directions, respectively. *t*_1_^0^ was the normal threshold stresses for PE/WPU composites and *t*_2_^0^, *t*_3_^0^ were the tangential threshold stresses for PE/WPU composites [24].

To predict the delamination extension under mixed-mode loading conditions, a second-order power law was utilized to characterize the interactions among the energies that contribute to failure in both normal and shear directions, including first and second shear:

(11) $${\left(\frac{G_{1}}{G_{1}^{0}}\right)}^{2}+{\left(\frac{G_{2}}{G_{2}^{0}}\right)}^{2}+{\left(\frac{G_{3}}{G_{3}^{0}}\right)}^{2}=1$$

where *G_i_* (i = 1, 2, 3) and *G_i_*^0^ (i = 1, 2, 3) represented the strain energy release rates and the critical fracture energy in three distinct directions, respectively [25,26].

During the fabrication of the FRPCs, changes in the resin modulus simultaneously led to variations in the resin tear strength and interfacial bonding strength. To investigate the relative influence of resin modulus, resin tear strength and interfacial bonding strength on the energy absorption of the FRPCs under different external loading conditions, the relative weight analysis model was applied based on the provided composite material data. The relative influence percentages of the following three attributes were calculated—*a* (modulus), *b* (tear strength) and *c* (interfacial bonding strength)—on the energy absorption under the following four external loading conditions, namely *E*_1_ (0.008 m/s), *E*_2_ (4.4 m/s), *E*_3_ (strain rate 1700 s^−1^, velocity at loading point 149.6 m/s) and *E*_4_ (approximately 400 m/s). The computational procedure included data standardization, correlation matrix calculation, eigenvalue and eigenvector analysis, orthogonal score computation, regression analysis, relative weight calculation and percentage determination, with all results retained to 4 significant figures.

To assess the influence of three correlated input variables (tensile modulus, tear strength and interlayer strength) on four distinct outcomes, this study employed a relative weight analysis based on principal component analysis. This approach addresses multicollinearity by transforming the original variables into orthogonal components, allowing the total explained variance in each outcome to be partitioned into non-overlapping contributions. The relative importance of each input variable can then be determined. Given the considerable differences in scale among the three inputs, the data were first standardized to ensure comparability. Sample means $\bar{x}$ and standard deviations ss were calculated using Equations (12) and (13), and the raw data were transformed into standardized variables $S_{x}$ via Equation (14), resulting in variables with a mean of zero and a standard deviation of one.

(12) $$\bar{x}=\frac{\sum x_{i}}{n}$$

(13) $$S_{x}=\sqrt{\frac{\sum {\left(x_{i}-\bar{x}\right)}^{2}}{n-1}}$$

(14) $$z_{i}=\frac{x_{i}-\bar{x}}{S_{x}}$$

(15) $$Z=\begin{array}{ccc} |Z_{a} & Z_{b} & Z_{c}| \end{array}$$

In the calculation of the correlation coefficients, since the data had been standardized with a mean of approximately 0 and a sample standard deviation of approximately 1, the expression for the correlation coefficient could be simplified from Equation (16) to Equation (17).

(16) $$R_{ab}=\frac{\sum \left(z_{a}-\bar{z_{a}})(z_{b}-\bar{z_{b}}\right)}{(n-1)S_{a}S_{b}}$$

(17) $$R_{ab}=\frac{\sum z_{a}z_{b}}{n-1}$$

After computing the relevant parameters, the correlation matrix *R*, eigenvector matrix *Q* and orthogonal fractional matrix *V* were obtained, which could capture the linear relationships among the three inputs.

(18) $$R=|\begin{array}{ccc} 1 & R_{ab} & R_{ac}\\ R_{ab} & 1 & R_{bc}\\ R_{ac} & R_{bc} & 1 \end{array}|$$

(19) $$Q=|\begin{array}{ccc} \upsilon_{1} & \upsilon_{2} & \upsilon_{3} \end{array}|$$

(20) V=ZQ

The standardized values of energy absorption were regressed on the orthogonal scores V through the following regression model, and the proportion of each influencing factor $IF_{x}$ under different external loads could be finally calculated. For each of the four standardized outcomes, a multiple linear regression model was fitted using the orthogonal scores from V as predictors. The regression results provide the variance explained by each principal component. Because the components are independent and each is a linear combination of the original variables, the explanatory power of each component can be traced back to the original inputs. This step yields the relative contribution of each input variable to the outcome, reflecting its unique influence after accounting for correlations among the inputs.

(21) $$Z_{E}=\beta_{1}V_{1}+\beta_{2}V_{2}+\beta_{3}V_{3}+\varepsilon ,\beta_{k}=\frac{{\sum Z_{E}\cdot V}_{k}}{\sum V_{k}^{2}}$$

(22) $$RW_{a}=\sum_{k=1}^{3}Q_{1k}^{2}\cdot \lambda_{k}\cdot \beta_{k}^{2}$$

(23) $$RW_{b}=\sum_{k=1}^{3}Q_{2k}^{2}\cdot \lambda_{k}\cdot \beta_{k}^{2}$$

(24) $$RW_{c}=\sum_{k=1}^{3}Q_{3k}^{2}\cdot \lambda_{k}\cdot \beta_{k}^{2}$$

(25) $$IF_{x}=\frac{RW_{x}}{RW_{a}+RW_{b}+RW_{c}}$$

## 3. Results

### 3.1. Fundamental Characteristics of Composites

#### 3.1.1. Dynamic Mechanical Analysis of WPU X

Blend WPU dispersions exhibiting low and high tensile strength in varying mass ratios to fabricate WPU films with systematically tuned mechanical properties—including tensile strength, elastic modulus, and shear strength. The resulting formulations are designated A through E in increasing order of elastic modulus. Five types of resin with different mix designs, designed with modulus of 9.5, 11.8, 13.3, 13.8 and 16.0 MPa, were used in this test.

**Figure 4:**
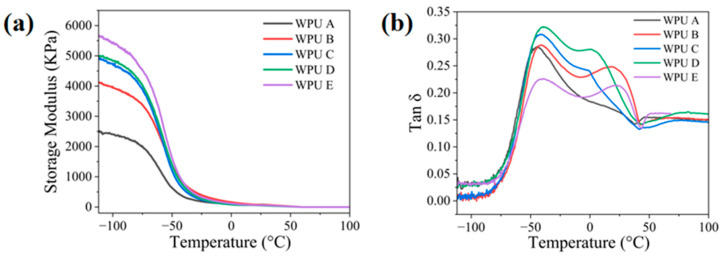
Dynamic mechanical analysis results of WPU X (**a**) temperature–storage modulus curves; (**b**) temperature–tanδ curves.

The resins were numbered in ascending order of modulus, and their basic properties were tested as presented in Table 4. The modulus range of the resins extended from 9.5 MPa to 16.0 MPa. An increase in the tensile elastic modulus of the resin is accompanied by corresponding increases in both its tensile strength and shear strength. The values of parameters *T*_g_^DMA^ and Tan δ exhibit close agreement, attributable to the application of the resin mixture method as shown in Figure 4.

#### 3.1.2. Quasi-Static Tensile Properties of PE/WPU Laminates

**Figure 5:**
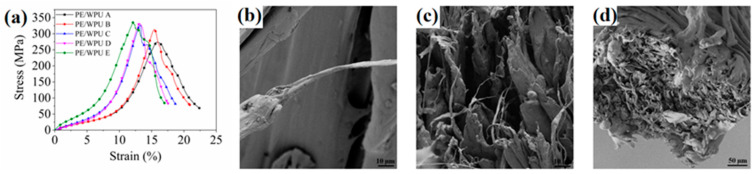
Experimental data of quasi-static tensile tests and SEM analysis of the fracture: (**a**) stress–strain curves of tensile properties of PE/WPU composites; (**b**–**d**) micromorphology of the fracture.

During the impact process, the yarn in the affected area experienced pull-out and tensile damage, while the yarn in the indirectly affected area underwent tensile deformation. This mechanism facilitated the absorption of the majority of the impact energy from the projectile. As shown in Figure 5a, the stress–strain curve of PE/WPU laminates illustrated that the mechanical properties of the matrix played a crucial role in determining the tensile properties of the composites. High modulus and strength of WPU contributed to the enhancement of the tensile strength of PE/WPU composites. The static tensile behaviour of PE/WPU composites could be categorized into three regions. In the initial tensile region, stress rapidly transmitted through fibres, causing original crimped yarn to gradually straighten. As the strain increased, yarn slippage occurred, leading to the degradation of the interface with the matrix. Subsequently, as strain and force continued to increase, the sample reached its ultimate tensile strength, leading to progressive fibre breakage and corresponding decrease in stress. Unlike the fracture behaviour of brittle materials, PE/WPU composites exhibited greater ductility, with tensile fracture occurring successively. Additionally, it was noted that higher modulus values of WPU could correspond to higher stress levels and a shorter time to reach the peak stress value.

**Figure 6:**
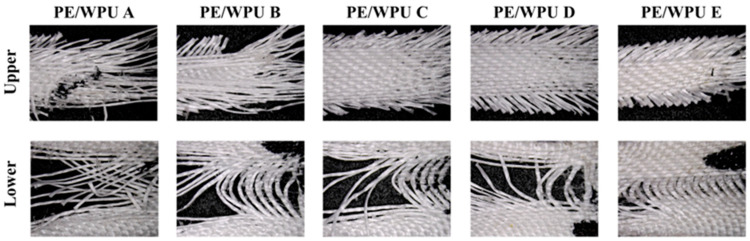
Optical microscope image of PE/WPU composites at the upper and lower end of the fabric fracture after quasi-static tensile test.

In Figure 5b, the damage morphology of a single strand was depicted, showing that the fibre had been pulled apart and the resin exhibited a pronounced necking phenomenon. Figure 5c,d illustrated the micromorphology of the representative areas at the fracture site. The composites gradually stretched to their limit, leading to sequential ductile fractures. This phenomenon also explained the observation in Figure 5a as follows: when tension reached its peak force, it did not plummet but instead declined gradually. Figure 6 depicted optical microscope images of the fibre pattern following tensile fracture. A comparison of PE/WPU A to E revealed that softer matrix exhibited a reduced binding force on the fibres during tensile loading, resulting in greater fibre dispersion upon pull-out. Conversely, the harder matrix contributed to better-bound fibres and a more concentrated breaking point.

#### 3.1.3. T-Peel Test Results of PE/WPU X Laminates

During the impact process, a significant amount of impact energy was absorbed as the stress propagated along the radial and the longitudinal directions of fibres, as well as between the laminates. Stress transfer in the warp and weft direction led to tensile failure of the original yarn and tensile deformation of secondary yarn. Additionally, stress transfer between layers resulted in partial energy absorption and blocks stress transition. The extent of layer damage hinged on the magnitude of the bonding force between the laminates.

**Figure 7:**
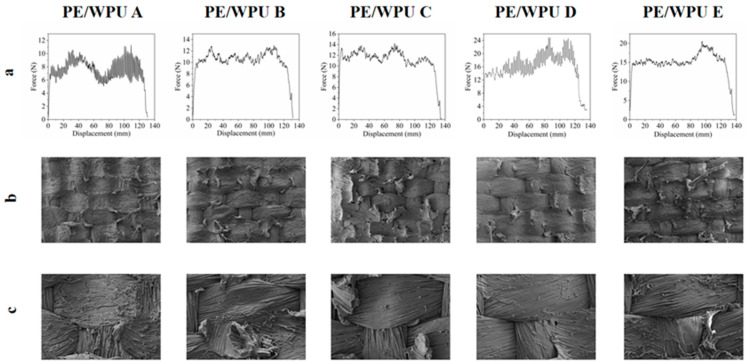
Experimental data of T-peel tests and SEM analysis of the stripped surface: (**a**) T-peel test results; (**b**) SEM analysis of overall morphology; (**c**) SEM analysis of partial morphology.

Figure 7 illustrated the load–displacement condition during the T-peel test, depicting the real-time interlayer adhesion strength measurement of PE/WPU X laminates. According to the load–displacement curve, the process of interface stripping could be divided into the following two distinct phases: initially, there existed a rapid increase in applied load, leading to the original stratification and crack formation. Subsequently, with full development of crack and delamination, the stripping force tended to stabilize until complete detachment occurred. Table 5 revealed that the specimen prepared with resin of higher modulus exhibited higher interfacial peeling strength. As shown in Figure 7a, PE/WPU A exhibited significant fluctuations, which were associated with its low resin modulus, superior toughness and weaker binding to fibres compared to other samples. Additionally, Figure 7b showed that during peeling process, fibres on the peeled surface of PE/WPU A underwent extensive deformation resulting in uneven overall force and deformation of surface WPU, leading to substantial fluctuations in the curve. During peeling process, once the resin was not tough enough to local the fibres, the fibres could then be affected and pulled out in any direction, and stress waves would not travel along the peeling direction. As illustrated in Table 5, it was evident that the average peel strength of PE/WPU D was 16.01 N and the average peel strength was 800.5 N/m—representing increases of 107.7% compared to PE/WPU A. This phenomenon could be attributed to higher resin modulus, which exhibited rigid brittle failure characteristics that contribute to the increased average peeling force and inter-layer peeling strength.

The energy absorption capability of composites was directly correlated with their impact resistance. The energy absorbed by each laminate during the peeling process could be calculated by integrating the force-displacement curve in Figure 7a. Table 5 provided a more scientific representation of the interfacial bond strength of the composites, including specific parameters such as average peeling force, average peeling strength and energy absorption. For instance, PE/WPU A had an energy absorption value of 0.97 J, while PE/WPU B, C, D, and E exhibited values of 1.36 J, 1.50 J, 2.13 J and 2.05 J respectively. However, as shown in the morphology in Figure 7c, the utilization of softer resin led to greater non-directional fibre deformation. In contrast, rigid and brittle resin demonstrated stronger binding forces on fibres during tensile deformation in the same direction, leading to an increased absorption capacity. Furthermore, an increase in resin modulus resulted in stress concentration, which facilitated the pulling out and breaking of more fibres, ultimately enhancing the overall absorption effect.

### 3.2. Characterization of Low-Speed Impact Resistance of PE/WPU X Laminates

#### 3.2.1. Yarn Pull-Out Test Results of PE/WPU X Laminates

**Figure 8:**
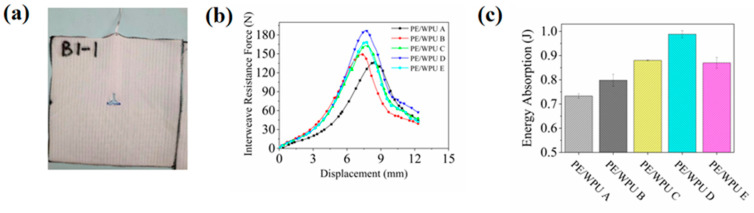
(**a**) Failure modes after yarn pull-out tests. (**b**) Pull-out test results. (**c**) Energy absorption during yarn pull-out tests.

Numerous studies have shown that incorporating resin into fibre can effectively absorb and dissipate energy through mechanisms such as fibre fracture, pull-out, and debonding within the resin matrix [27,28,29]. The results of the pull-out tests, illustrated in Figure 8b, demonstrated performance differences among the PE/WPU A, PE/WPU B, PE/WPU C, PE/WPU D and PE/WPU E composites. It was noteworthy that force-displacement curves exhibited the following two distinct stages: the yarn de-crimping stage and the yarn translation stage. Initially, as the pulling force increased, the yarn gradually de-crimped due to static friction at yarn crossing point and between adjacent yarn. This constraint limits the pulling behaviour until surpassing the constraint force transitions into a second stage where resistance primarily arises from perpendicular and parallel adjacent yarns. Simultaneously, gradual degradation of the surrounding fabric structure led to a decline in pulling force. The displacement-force profiles derived from the yarn pull-out test (Figure 8b) further illustrated these dynamics. Notably, under identical conditions, fabrics with higher modulus demonstrated significantly better pull-out forces compared to those with lower modulus. For PE/WPU compositions labelled as PE/WPU A, B, C, D and E peak pull-out forces were recorded at 136.1 N, 149.2 N, 163.1 N, 186.9 N and 168.7 N respectively.

Energy absorption capacity of the fabric directly influenced its impact resistance. Taking the same displacement value (12.3 mm) as displacement integral of the force displacement curve in Figure 8b, the energy absorbed by the composites could be obtained, as shown in Figure 8c. Compared with the force displacement curve, Figure 8c more scientifically reflected the ability of composites to absorb energy during the whole extraction process. The energy absorption value of PE/WPU A was 0.73 J. With the increase in modulus, energy absorption values of PE/WPU B, C, D and E were 0.80 J, 0.88 J, 0.99 J and 0.87 J respectively, representing increases of 8.9%, 20.1%, 34.8% and 18.7% compared to PE/WPU A. Composites with higher modulus had an overall improvement in extraction energy compared to the composites with the lowest resin modulus. Combining these two sets of data led to the conclusion that resin-impregnated fibre fabrics with higher modulus had better fibre pull-out capacity and energy absorption performance, thus indicating preferable low-speed impact resistance [30]. However, we noticed that the series of data were not a simple linear relationship. When the modulus reached maximum, the performance of PE/WPU E was lower than that of PE/WPU D. This had triggered more thinking and research.

**Figure 9:**
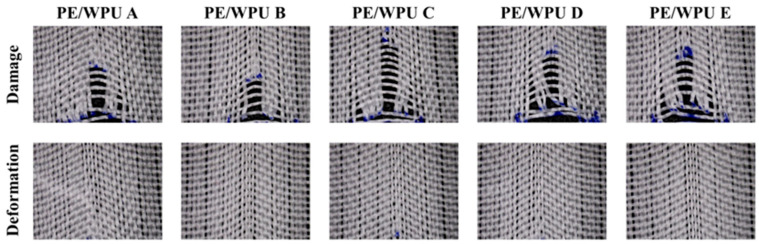
Optical microscope images of the PE/WPU composites at the notch and force deformation of fabrics after yarn pull-out tests.

The damage modes of the fabric after the yarn pulling tests were illustrated in Figure 8a. Additionally, Figure 9 showed optical micrograph of the damaged part of the fabric at the gap, along with the optical micrograph of the fabric above the gap. The whole specimen could be divided into the following three parts: the main influence area, the sub-influence area and the undeformed area. The specimens prepared with resin of high modulus had stronger binding force to the fibres. In the process of yarn pulling, on the one hand, the strong binding force increased the friction between the drawn fibres and the direct contact fibres. On the other hand, it would cause the phenomenon of stress concentration, reducing numbers of fibres in the sub-affected area. The yarn pulling performance of the specimen resulted from the interplay of the aforementioned two influencing factors, which could account for the observed trend in energy absorption values—initially increasing and subsequently decreasing with the rise in resin modulus.

#### 3.2.2. Low-Velocity Drop Hammer Results of PE/WPU X Laminates

**Figure 10:**
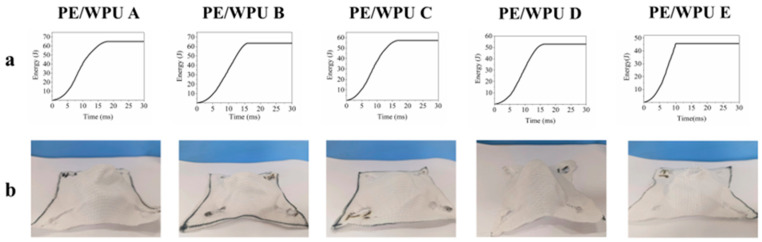
(**a**) Energy absorption graphs PE/WPU composites; (**b**) corresponding failure modes after dynamic impact tests.

In order to investigate the influence of resin modulus on the energy absorption properties of fabric during low-velocity impacts, dynamic impact tests were conducted on PE/WPU X composites. Figure 10a illustrated the results of dynamic impact tests, showing energy absorption over time and the failure modes of five different composites. During the dynamic impact process, PE/WPU X composites underwent the following three stages: de-crimping, pull-out and destruction. Upon contact with the drop hammer, the fabric was initially de-crimped and then stretched to its elastic limit. The pull-out of the fibre resulted in tensile stress and absorption of impact energy. At the same time, this behaviour was transmitted through stress transfer centred at the point of contact towards all four sides. It was noted that as the distance from the point of impact increased, there was a decrease in observable pull-out behaviour in the yarn [31]. Ultimately, once deformation reached its elastic limit zone, damage occurred within the fibre.

It could be observed from Table 6 that the peak force of PE/WPU A, PE/WPU B, PE/WPU C, PE/WPU D, and PE/WPU E increased from 2.56 kN to 2.97 kN respectively with the increase in resin modulus. This increase was attributed to the stress concentration caused by the high modulus of the resin. The energy absorbed by these samples was observed to decrease initially, followed by an increasing trend, with values of 63.5 J, 57.2 J, 60.9 J, 65.0 J and 75.7 J respectively. The failure mode diagram in Figure 10b clearly illustrated that as the focus shifted from PE/WPU A to PE/WPU D, the affected area of each sample continued to decrease due to the reduction in stress wave transmission speed associated with the increasing resin modulus. Compared to materials prepared with a higher matrix modulus, PE/WPU A exhibits the largest deformation area, the lowest peak force, and the smallest deformation degree. However, it absorbs more energy during low-speed impact tests due to its softer matrix, which exerts less binding force on the fibres, allowing for greater fiber deformation and consequently higher energy absorption [32]. Upon further increasing the resin modulus to PE/WPU E, complete penetration occurred in a minimal amount of time, resulting in a minimal affected area and energy absorption value.

Given that the total energy of the dynamic shock is determined, softer matrix and lower binding force resulted in greater tensile behaviour exhibited by sub-yarns not in direct contact with the impactor. Consequently, this enhanced the energy absorption of composites. Similarly, higher modulus of resin would contribute to stronger peak forces and increased energy absorption density per unit area. However, as the modulus of resin increased, an inverse relationship emerged between energy absorption density per unit area and impact area. This trend remained consistent within a specific range of resin modulus. When the resin modulus surpassed the threshold, an excessively high modulus could cause stress concentration, leading to premature penetration of the specimens. This occurred before the stress was effectively transferred to fibres not in direct contact with the impactor, ultimately resulting in lower final energy absorption values.

### 3.3. Characterization of High-Speed Impact Properties of PE/WPU X Laminates

#### 3.3.1. HSR (High-Strain Rate) Tensile Properties of PE/WPU X Laminates

**Figure 11:**
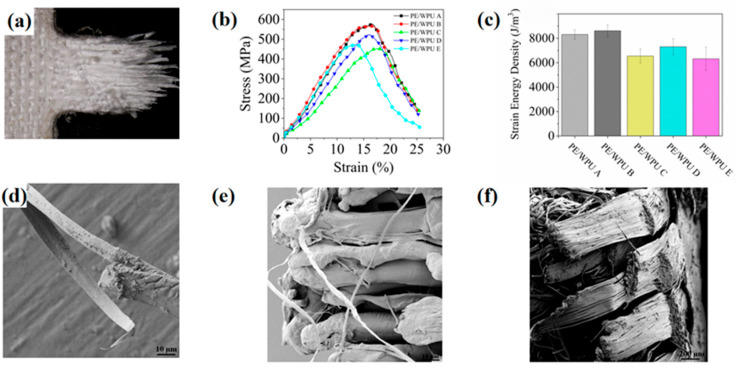
Damage of the characterization after HSR test and experimental data: (**a**) optical microscope photos of fracture; (**b**) stress–strain curves; (**c**) strain energy density; (**d**–**f**) micromorphology of damage at fracture after HSR tensile test.

During the HSR test, it was observed that strain rate of PE/WPU X laminates exhibited a rapid increase in the initial stage (strain < 5%), followed by a tendency to stabilize at approximately 1700 s^−1^. The internal wave speed of the device reached 5100 m/s, effectively reflecting the high-speed impact resistance of composites. Figure 11a depicted the general fracture morphology of samples after HSR test. The aligned fibres along the tensile direction did not cleanly break, possibly due to the tendency of composites to fracture at their defects under HSR conditions. Additionally, near the fracture, a noticeable deviation at a large angle was observed in secondary yarn perpendicular to the tensile direction. However, this phenomenon was limited to a small area and did not significantly affect other regions. This phenomenon could be attributed to the HSR conditions, which caused a delayed transfer of stress waves between fibres and resin, as well as at the interfaces between laminates [33]. As depicted in Figure 11b, it was evident that under HSR condition, resin with a lower tensile elastic modulus resulted in a higher maximum stress value. The integration of stress with strain allowed for the determination of strain energy density (shown in Figure 11c). The strain energy densities of patterns PE/WPU A and PE/WPU B were significantly greater than those of the resins with higher modulus. Table 7 presented peak force and strain energy density of the patterns. Notably, the strain energy density values of patterns A and B were similar to each other, and significantly superior to those of patterns C, D and E, respectively. Furthermore, Table 7 indicated that the strain energy density of PE/WPU B was 8616.9 J/m^3^, which was 31.6%, 17.9%, and 36.4% higher than that of PE/WPU C, D and E, respectively. Resin with a lower tensile elastic modulus exhibited better resistance to high-speed impacts. The morphologies shown in Figure 11d–f were obtained by SEM.

By comparing Figure 5b and Figure 11d, it was evident that the deformation of resin in the HSR tensile test was less than that observed in the static tensile test. Figure 11e illustrated that the discontinuity in the experimental process or defects within the fibres themselves led to fibre fibrillation. Consequently, under pure tensile load, shear stress was generated in the plane perpendicular to the tensile direction. When the tensile load was sufficiently high, even though the resin remained intact, shear stress could overcome transverse bonding force and produced longitudinal splitting. If a crack deviated slightly from the fibre axis, it would eventually propagate through the fibre and cause fracture as an inevitable outcome. Under a combination of tensile load and partial shear stress, neat step-shaped fracture morphology was observed in HSR sample as shown in Figure 11f.

#### 3.3.2. Ballistic Impact Properties of PE/WPU X Laminates

**Figure 12:**
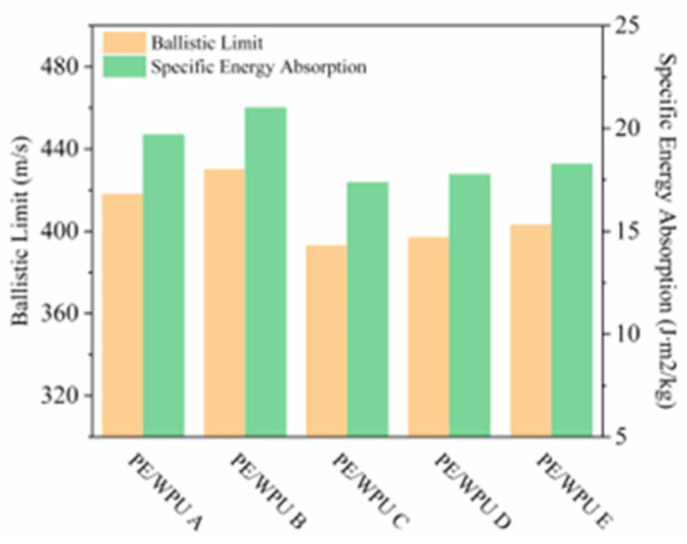
Ballistic performances of PE/WPU laminates: V50 and SEA.

As illustrated in Figure 12, the ballistic test results of PE/WPU composites exhibited a characteristic S-shaped decline trend. The laminates prepared by lower modulus resins had better anti-elasticity, among which PE/WPU B had the best anti-elasticity, with V50 reaching 430 m/s and SEA reaching 21.01 J∙m^2^/kg (as shown in Table 8). This observation indicated that laminates with softer matrices retained sufficient toughness that could ensure optimal interfacial bonding strength and effective stress transfer capability, thereby demonstrating superior ballistic performance, which could enable extensive deformation and fibre pull-out. Concurrently, weaker interlayer bonding could result in layer-by-layer stratification, thereby allowing more fibres to absorb kinetic energy from the projectile. In contrast, resins with high modulus could induce stress concentration and crack phenomena. Although stress waves propagated faster in laminates of high stiffness, their progression was impeded at cracks, ultimately diminishing the anti-penetration performance.

**Figure 13:**
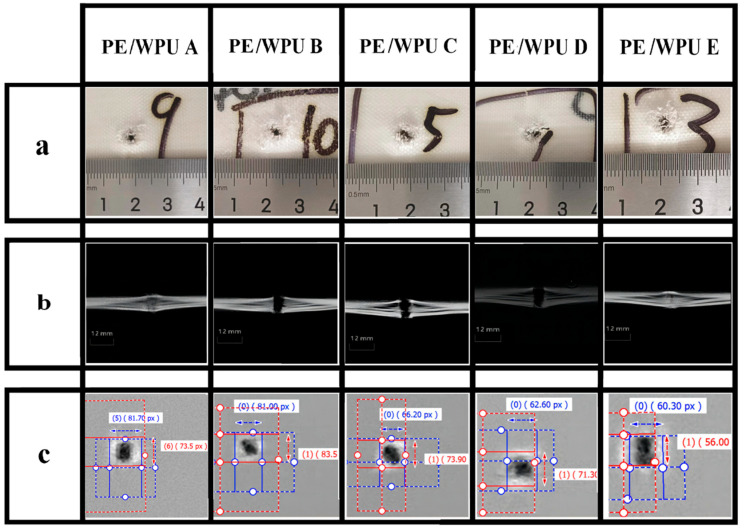
Characterization of the target plate penetration area: (**a**) picture of the impact face damage; (**b**) CT scan of interlayer destruction; (**c**) digital radiography of impact face.

To elucidate ballistic mechanisms, digital radiography scans were performed on the impact surfaces of specimens (Figure 13a), revealing similar damage morphologies but varying size of the damage areas. The primary failure mode on the impact surface of the laminates was mainly in the form of compression-shear. Notably, fabricated with lower tensile modulus resins, PE/WPU A and PE/WPU B displayed significantly broader impact surface damage areas while PE/WPU D and PE/WPU E specimens assumed less evident compressive-shear damage. The laminates which were distinguished by higher stiffness and modulus, especially PE/WPU E, manifested more pronounced compressive-shear failure patterns. Upon impact, high-velocity projectiles initially generated compressive stress waves along the thickness direction, exhibiting gradient attenuation within the laminates. The projectile then continued to penetrate, inducing combined compressive and shear damage.

During projectile penetration, compressive-shear failure dominated as the primary failure mechanism during the initial stage, transitioning to tensile-shear failure in the terminal phase. The kinetic energy of the projectile was progressively absorbed and dissipated through the following three synergistic mechanisms: fibre pull-out from the resin, fibre fracture, and interlayer delamination. In the compression-shear zone, PE/WPU A and PE/WPU B had less constraint on fibres, enabling more fibre deformation in secondary affected zones during impact. This forced prolonged velocity attenuation of the projectile over an extended interaction range, resulting in extensive fibre pull-out, fracture, and progressive delamination, contributing to higher energy absorption values [34]. Conversely, laminates with high-modulus exhibited rapid stress transfer rates but poor toughness, causing stress concentration and propagation of cracks that compromised energy absorption efficiency [35].

In the tension-shear zone, under equivalent fibre strength conditions, laminates demonstrating more complete fibre stretching exhibited better ballistic resistance. Laminates prepared with softer resins such as WPU A and WPU B facilitated fibre-bending deformation and coordinated matrix deformation. This synergistic effect promoted combined deformation and fracture of secondary-zone yarns, so as to increase kinetic energy absorption. In contrast, more rigid laminates accelerated stress transfer rates, resulting in localized fibre fracture at direct impact points without significant secondary-zone fibre pull-out, ultimately degrading ballistic performance [36].

Finite element simulations using ABAQUS were conducted to validate experimental findings, which could analyse damage mode and stress distribution during projectile penetration. The PE/WPU X finite element model incorporated material parameters which were obtained from experimental measurements and literature references. The residual projectile velocities approached 0 m/s near penetration thresholds were chosen as simulation V50 results. The finite element simulations performed at varying impact velocities showed excellent consistency with experimental V50 ballistic limit values.

PE/WPU laminates exhibited compressive-shear failure during the initial penetration phase, transitioning to delamination and fibre tensile failure in later stages. While different modulus of the matrix did not alter the fundamental failure sequence, matrix mechanical properties significantly influenced damage evolution and mechanistic progression. Composites of softer matrix could exhibit limited compressive-shear damage and mild delamination, transitioning to extensive tensile failure during late penetration stages [37]. Conversely, rigid matrix composites demonstrated stress concentration characteristics, sustaining severe compressive-shear damage initially and substantial delamination subsequently, both of which hindered stress propagation and deteriorated anti-penetration performance.

**Figure 14:**
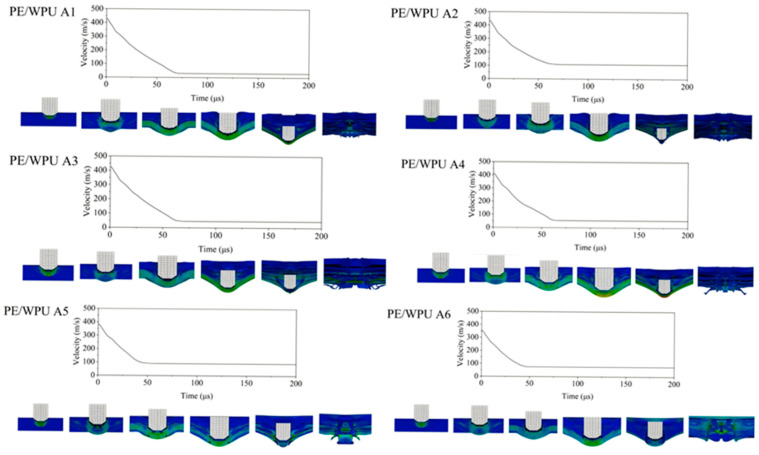
Simulation diagrams of the penetration results of the same material under different interlayer bonding strengths.

As shown in Figure 14, the simulation results also further confirmed this outcome. The Abaqus simulation results reveal the regulation mechanism of interlaminar bonding strength on the dynamic response behaviour of the material system under ballistic impact. The figure presents the projectile velocity–time curves and penetration processes for six groups with different interlaminar bonding strengths, labelled from PE/WPU A1 to A6. All results as shown in Table 9 were obtained under complete perforation conditions, with the difference in initial projectile velocity between perforation and non-perforation cases kept within 10 m/s. Each curve exhibits the following typical impact response characteristics: the projectile velocity decreases rapidly at the initial moment of contact and completes perforation within 50–100 ms.

A comparison of the curves and experimental results indicates that a moderate level of bonding strength—specifically, lower bonding strength—yields superior impact resistance. Under low bonding strength conditions (A1–A3), the projectile velocity decreases more substantially during impact, and the curves exhibit a prolonged energy dissipation plateau. This suggests that appropriate interfacial bonding allows the material system to dissipate substantial impact energy through mechanisms such as interlaminar debonding and interfacial sliding, thereby avoiding stress concentration and localized failure. As bonding strength progressively increases (A4, A5), the enhanced interfacial bonding restricts relative interlayer displacement, concentrating impact energy transfer to the material matrix. This results in less effective velocity deceleration and higher residual velocity. When bonding strength reaches its highest level (A6), although the projectile velocity decreases rapidly in the initial stage, a pronounced rebound or secondary acceleration appears in the subsequent curve. This indicates that excessively strong interfacial constraints force impact energy to be released through brittle failure of the matrix or fibres, ultimately reducing the system’s overall energy absorption efficiency. In summary, the simulation results demonstrate that lower bonding strength improves the ballistic impact resistance of the material system. The underlying mechanism lies in the ability of weaker interfaces to induce controlled interlaminar debonding and sliding, serving as an effective energy dissipation pathway.

#### 3.3.3. The Results of Relative Weight Analysis

The relative weight analysis was conducted on the effects of resin modulus, resin tear strength, and interfacial bonding strength on the energy absorption value of composites under low-velocity impact. The results indicated that in yarn pull-out tests, the contributions of resin modulus, resin tear strength, and interfacial bonding strength to the energy absorption value were 40.25%, 47.45%, and 12.30%, respectively. In low-velocity drop-weight impact tests, the corresponding contributions were 51.70%, 27.30%, and 21.00%. Analysis of the results suggests that under low-velocity impact loading, the resin should possess sufficient stiffness to fix the fibres and transfer stress. Under low-speed loading conditions, the material does not reach its ultimate state; instead, fibre deformation mechanisms such as unbending and stretching dominate, with no significant fibre fracture observed. Consequently, the demand for resin tear strength and interfacial properties in terms of energy absorption is relatively low. Therefore, under low-velocity impact, the resin modulus exerts the greatest influence on energy absorption among the coupled factors. In the selection of raw materials for composites designed to resist low-velocity impact, particular attention should be paid to the tensile elastic modulus of the resin.

A relative weight analysis was performed to evaluate the influence of resin modulus, resin tear strength, and interfacial bonding strength on the energy absorption of composites under high-velocity impact. The results show that in the HSR test, the contributions of resin modulus, resin tear strength, and interfacial bonding strength to the total energy absorption were 18.50%, 48.60%, and 32.80%, respectively. In ballistic tests, the corresponding contributions were 8.76%, 48.80%, and 42.40%. Analysis of the results indicates that under high-velocity impact loading, interfacial bonding strength and tear strength of resin are the primary factors. In such high-rate loading conditions, composites absorb energy through multiple mechanisms, including fibre unbending, stretching, fracture, and delamination of fabric layers. Therefore, it is essential to consider the shear resistance strength of the composites and the interlayer bonding strength of laminates, corresponding to shear-induced damage and interlaminar delamination in the target plate during ballistic penetration. Thus, both tear strength of the resin and interfacial bonding strength of the composites should be taken into account during the material design and manufacturing process.

## 4. Conclusions

In this study, PE/WPU X laminates with different resin modulus were prepared to investigate the influence of matrix properties on the impact resistance of FRPCs and the proportion attributable to each coupling factor. The experiments included quasi-static experiments, yarn pull-out tests, low-velocity impact tests, HSR tensile tests, ballistic impact tests, numerical simulation and relative weight analysis. The results indicated that resin modulus accounted for the largest weight among all coupling factors. With increasing the resin modulus, the FRPCs transitioned from being soft and tough to strong and rigid, which subsequently affected their resistance to both low-speed and high-speed impacts.

In low-speed loading environments, the resin modulus had the greatest weight in the energy absorption value. FRPCs prepared with higher tensile elastic modulus resin exhibited enhanced mechanical properties and improved low-speed impact resistance. This improvement was attributed to the increased friction between yarns due to a higher modulus of the resin, which facilitated greater energy absorption through fibre pull-out. Although stress concentration might occur, inhibiting fibre movement and stress wave transmission in such cases, the relatively low loading rate allowed sufficient time for stress wave propagation, rendering any potential impact negligible.

In high-speed impact environments, tear strength of resin and interlayer bonding strength of laminates took up a more significant proportion of energy absorption. The ballistic performance of PE/WPU X initially increased before exhibiting a decreasing trend. This suggested that an elastomer required a certain stiffness and interlaminar bonding strength; a softer ductile matrix demonstrated better ballistic properties by quickly transferring stress during the penetration process. This allowed for wider range of deformation and fibre pull-out while reducing delamination damage through weak interlayer binding forces, thereby enabling more fibres to absorb kinetic energy from projectiles. In contrast, rigid brittle resins led to stress concentration, which impeded late transmission of stress waves, resulting in significant delamination damage and reduced elastic properties.

## Figures and Tables

**Table 1:** Mechanical properties of the modelling clay [11,16].

*E* (GPa)	*σ* (GPa)	*Ν*	*ρ* (kg/m^3^)	*K* (MPa)	*N*
5.347 × 10^−3^	1 × 10^−5^	0.49	1539	0.3609	0.1649

**Table 2:** Mechanical properties of PE/WPU X laminates.

***E*_1_ (GPa)**	***E*_2_ (GPa)**	***E*_3_ (GPa)**	***G*_12_ (GPa)**	***G*_13_ (GPa)**	***G*_23_ (GPa)**	***ν*_12_**	***ν*** **_13_**	***ν*** **_23_**	***ρ* (kg/m^3^)**
50.00	50.00	22.00	0.77	5.34	5.34	0.25	0.33	0.33	1053
***X_T_* (MPa)**	***X* _C_(MPa)**	***Y*_T_ (MPa)**	***Y*_C_ (MPa)**	***S*_12_ (MPa)**	***S*_13_ (MPa)**	***S*_23_ (MPa)**			
650	300	650	300	120	220	220			

**Table 3:** Interface properties of PE/WPU X.

Samples	*Enn* (MPa)	*Ess* (MPa)	*Ett* (MPa)	*t*_1_^0^ (MPa)	*t*_2_^0^ = *t*_3_^0^ (MPa)	*G*_1_^0^ (N/mm)	*G*_2_^0^ = *G*_3_^0^ (N/mm)
A1	2	1	1	6.41	3.20	0.67	1.34
A2	50	25	25	6.41	3.20	0.67	1.34
A3	100	50	50	6.41	3.20	0.67	1.34
A4	200	100	100	6.41	3.20	0.67	1.34
A5	400	200	200	6.41	3.20	0.67	1.34
A6	800	400	400	6.41	3.20	0.67	1.34

The *t*_1_^0^ values of the PE/WPU X laminates were regarded as equivalent to the tensile strength of PE/WPU X. The *t*_2_^0^ and *t*_3_^0^ were considered equivalent to half of the *t*_1_^0^. The *G*_2_^0^ and *G*_3_^0^ values were considered to be twice the *G*_1_^0^ values.

**Table 4:** Thermal and mechanical properties of WPU X.

Samples	Tensile Strength (MPa)	Strain at Break (%)	Tensile Modulus of Elasticity (MPa)	*T*g^DMA^ (°C)	Tan δ (Max)	Tear Strength (kN/m)
WPU A	9.5	1387.6	8.02	−39.42	0.32	5.01
WPU B	11.8	1375.4	10.81	−41.77	0.31	12.60
WPU C	13.3	1552.7	10.92	−40.98	0.29	16.34
WPU D	13.9	1334.2	11.79	−39.39	0.23	18.97
WPU E	16.0	1419.3	19.47	−44.37	0.29	22.00

**Table 5:** T-peel test results of PE/WPU composites.

Samples	Average Peeling Force (N)	Average Peeling Strength (N/m)	Energy Absorption (J)
PE/WPU A	7.71	385.5	0.97
PE/WPU B	10.81	540.5	1.36
PE/WPU C	11.68	584.0	1.50
PE/WPU D	16.01	800.5	2.13
PE/WPU E	15.77	788.5	2.05

**Table 6:** Low-velocity impact properties of PE/WPU composites.

Sample Designation	Peak Force (kN)	Absorbed Energy (J)
PE/WPU A	2.56	63.5
PE/WPU B	2.61	57.2
PE/WPU C	2.68	60.9
PE/WPU D	2.77	65.0
PE/WPU E	2.97	75.7

**Table 7:** HSR tensile test results of PE/WPU composites.

Sample Designation	Peak Stress (MPa)	Strain Energy Density (MJ/m^3^)
PE/WPU A	577.3	8312.5
PE/WPU B	566.9	8616.9
PE/WPU C	452.9	6548.4
PE/WPU D	523.5	7307.5
PE/WPU E	472.9	6318.0

**Table 8:** Ballistic impact results of PE/WPU composites.

Samples	Areal Density (kg/m^2^)	Ballistic Limit (m/s)	SEA (J∙m^2^/kg)
PE/WPU A	4.88	418	19.69
PE/WPU B	4.84	430	21.01
PE/WPU C	4.89	393	17.37
PE/WPU D	4.88	397	17.76
PE/WPU E	4.89	403	18.27

**Table 9:** Simulation experiment results.

Sample Designation	V50 (m/s)
PE/WPU A1	426
PE/WPU A2	425
PE/WPU A3	415
PE/WPU A4	410
PE/WPU A5	390
PE/WPU A6	355

## Data Availability

The datasets used and/or analyzed during the current study are available from the corresponding author on reasonable request.

## Abbreviations

The following abbreviations are used in this manuscript:

FRPCs	Fibre-reinforced polymer composites
WPU	Waterborne polyurethane
UHMWPE	Ultra-high molecular weight polyethylene
HSR	High-strain rate
